# Polyphenols as Key Players for the Antileukaemic Effects of Propolis

**DOI:** 10.1155/2014/371730

**Published:** 2014-03-19

**Authors:** Murtala B. Abubakar, Wan Zaidah Abdullah, Siti Amrah Sulaiman, Boon Suen Ang

**Affiliations:** ^1^Human Genome Centre, School of Medical Sciences, Universiti Sains Malaysia, 16150 Kubang Kerian, Kelantan, Malaysia; ^2^Department of Physiology, College of Health Sciences, Usmanu Danfodiyo University, PMB 2254, Sokoto, Nigeria; ^3^Department of Haematology, School of Medical Sciences, Universiti Sains Malaysia, 16150 Kubang Kerian, Kelantan, Malaysia; ^4^Department of Pharmacology, School of Medical Sciences, Universiti Sains Malaysia, 16150 Kubang Kerian, Kelantan, Malaysia; ^5^Department of Physiology, School of Medical Sciences, Universiti Sains Malaysia, 16150 Kubang Kerian, Kelantan, Malaysia

## Abstract

Propolis (a bee product) which has a long history of medicinal use by humans has attracted a great deal of research interest in the recent time; this is due to its widely reported biological activities such as antiviral, antifungal, antibacterial, anti-inflammatory, antioxidant, and anticarcinogenic properties. Crude form of propolis and its phenolic contents have both been reported to exhibit antileukaemic effects in various leukaemia cell lines. The ability of the polyphenols found in propolis to arrest cell cycle and induce apoptosis and differentiation in addition to inhibition of cell growth and proliferation makes them promising antileukaemic agents, and hence, they are believed to be a key to the antileukaemic effects of propolis in different types of leukaemia. This paper reviews the molecular bases of antileukaemic activity of both crude propolis and individual polyphenols on various leukaemia cell lines, and it indicates that propolis has the potential to be used in both treatment and prevention of leukaemia. This however needs further evaluation by *in vitro*, *in vivo*, and epidemiological studies as well as clinical trials.

## 1. Introduction

### 1.1. Propolis

Propolis also known as bee glue is a dark resinous substance collected by the worker bees from various plant exudates which they use to fill spaces and to coat walls of the hive for protection [1, 2]. It has a long history of medicinal use by humans [3] and has captured the attention of many researchers as a result of its antiviral, antifungal, antibacterial, anti-inflammatory, antioxidant, and anticarcinogenic properties [4–20].

Although the constituents of the various types of propolis vary largely depending on their plant origin, their biological activities reported in the literature are however very similar. For instance, Bogdanov has reviewed that the two main propolis varieties found on the market are* Baccharis* and poplar, and they both possess similar biological activities even though they have different active ingredients (Table 1) [21]. Based on the plant origin where bees collect exudates for the formation of propolis, poplar and* Baccharis* propolis represent propolis from temperate and tropical regions, respectively, [22]. Propolis is seldom used in its crude form, but rather its concentrated ethanolic extracts obtained by extraction with 70% ethanol are used for medicinal purposes [23]. A recent review by Kurek-Górecka and colleagues has reported that about 300 compounds have been isolated from propolis and notable groups among them are phenolic acids, flavonoids, terpenes, lipid-wax substances, beeswax, bioelements, and many other constituents like vitamins, proteins, amino acids, and sugars [23]. In terms of quantity and type, phenolic compounds (polyphenols) make up the most numerous group of compounds found in propolis [23].

### 1.2. Polyphenols

Polyphenols (phenolic compounds) are a group of metabolites that are widely distributed in the plants and found in abundance in a variety of fruits and vegetables as well as in honey, propolis, and royal jelly [21, 24, 25]. The mechanism of anticancer activities of dietary polyphenols has been previously reviewed [26]. The biological activities of polyphenols depend on their structure [27, 28] and based on our literature search, flavonoids and phenolic acids are the two most extensively studied groups of polyphenolic contents of propolis with respect to antileukaemic activity. These two important groups of compounds are briefly discussed below.

#### 1.2.1. Flavonoids and Phenolic Acids

Flavonoids constitute a chemical category of polyphenols with a wide variety of structure and biological activities [29]. Flavonoids are tricyclic compounds consisting of two phenolic rings designated as A and B and ring C containing a pyrane moiety, bounded through a C6-C3-C6 unit comprising 15 carbon atoms [30]. The flavonoids found in propolis are aglycones of glycosidic components of plants [23]. About 9 flavonoids were isolated from ethanolic extracts of polish propolis [31]. Based on their chemical structure, the major classes of flavonoids include flavanols, flavonols, flavones, isoflavones, anthocyanidins, dihydroflavonols, and chalcones [26, 30]. Flavonoids from dietary sources vary according to positions of hydroxyl, methoxy, and glycosidic side groups as well as in the combination between A and B rings [29].

Phenolic acids are commonly categorized into two major groups which mostly exist in the hydroxylated form, namely, hydroxybenzoic (containing seven carbon atoms-C6-C1) and hydroxycinnamic acids (containing nine carbon atoms-C6-C3) [26]; and these polyphenols and their derivatives represent the major phenolic acids found in Polish poplar type of propolis. Notable among the hydroxybenzoic derivatives are* p*-hydroxybenzoic acid,* p*-methoxybenzoic acid, and gallic acid. On the other hand, the commonly encountered cinnamic acid derivatives in Polish poplar type of propolis comprised caffeic acid,* p*-coumaric acid, isoferulic acid, and 3,4-dimethoxycinnamic acid [23].

#### 1.2.2. Absorption and Metabolism of Flavonoids and Phenolic Acids

It is fundamental to understand the biological processes that ensue after oral ingestion of flavonoids. There is no doubt that several* in vitro* studies have demonstrated the biological activities of flavonoids under a variety of disease conditions including leukaemia and other malignancies. However, there is paucity of information on the current understanding of pharmacokinetics and pharmacodynamics of most flavonoids in humans with only a few like quercetin having been extensively studied [29, 30]. Therefore, in order to fully incorporate the new knowledge of antileukaemic property of propolis into subject of nutrition and medicine, the following points must be delineated as suggested by Heim and colleagues [29]: (a) the degree of absorption in relation to structure, (b) pharmacokinetics in human subjects, (c) delineating the metabolites of its phenolic contents, and (d) structure activity relationship and health effects of the phenolic metabolites. Data on absorption and metabolism of flavonoids are very complex and have been extensively reviewed elsewhere [29]. In spite of this, it is imperative to highlight some salient points about the pharmacokinetics of flavonoids. In an* in vivo* study in rats conducted by Bravo et al., it was demonstrated that a little fermentation of catechin and tannic acid occurred in the gut by gut flora, and this was followed by excretion of less than 5% of these compounds in the faeces unchanged, thus, suggesting that some absorption had taken place [32]. Another study by Spencer et al. has also demonstrated hydrolysis and absorption of luteolin-7glucoside, kaempferol-3-glucoside, and quercetin-3-glucoside in rats' small intestine, thus, supporting the presence of *β*-glucosidase activity [33]. On the other hand, some human studies have reported that flavonoids are absorbed and metabolized following oral intake [34], whereas other studies have suggested that flavonoids are poorly absorbed and that not a significant portion of them reach the general circulation unchanged [35]. It is also important to note that the majority of investigations on pharmacological activities of flavonoids in humans mainly evaluated the metabolism of individual flavonoids used at pharmacological doses instead of estimated amounts present in dietary sources [34, 35] between 23 [36] and 170 mg daily [37]. Thus, making inferences in the results of these studies might be unfitting to describe the absorption and metabolism of dietary flavonoids [30] including those present in propolis.

### 1.3. Major Cellular Signaling Pathways and Mechanisms of Action of Polyphenols on Cancer and Leukaemic Cell Lines

#### 1.3.1. Induction of Apoptosis

Apoptosis or programmed cell death is a different type of cell death characterized by both morphological and biochemical changes in the form of cell shrinkage, DNA fragmentation, membrane blebbings, chromatin condensation, and loss of adhesion and rounding [38, 39]. Under physiological conditions, apoptotic cell death is accomplished via two different mechanisms, namely, the intrinsic pathway which is mediated via the mitochondria-dependent mechanism and the extrinsic pathway which is associated with death receptor mechanism [40, 41]. The p53, IAPs, and caspase and Bcl families are some of the major proteins that regulate apoptotic cell death. The p53 gene regulates both cell cycle and apoptosis [42]. It induces cell death by stimulating the release of mitochondrial cytochrome c and Smac/DIABLO (mediated by Bcl-2 family) both of which are believed to be responsible for the regulation of caspases activity that eventually causes the apoptotic cell death [43, 44]. Bcl-2 family proteins are mainly found in the mitochondrial membrane and are made up of antiapoptotic (Bcl-2, Bcl-X_L_, Mcl-1, and Bfl-1/A1) and proapoptotic (Bax, Bek, Bad, Bid, Bim, Puma, and Noxa) molecules. These important proteins play a vital role in mitochondria-mediated apoptotic cell death [41, 43]. The extrinsic pathway is regulated by CD95 and TRAIL which when activated bind to membrane-specific receptors such as death receptors (DRs).

The resultant interaction leads to formation of DISC which by a way of triggering the activation of caspases results in death of tumor cells without inducing inflammation or causing tissue damage [40]. IAPs are a family of molecules that exert inhibitory effects on activity of caspases 3, 7, and 9 [43]. Knowledge of molecular events in apoptosis has broadened the understanding of efficient cancer chemotherapy, and many of the reported anticancer agents destroy malignant cells by causing apoptotic cell death [39].

Flavonoids have been reported to induce apoptotic cell death in various cancer cell lines including a variety of leukemic cell lines, but sparing the normal noncancer cells. They achieve this by a number of mechanisms which include the following.

- Inhibition of DNA topoisomerase I/II activity: topoisomerases are important enzymes that play a vital role in maintaining topology of DNA during the process of DNA metabolism, that is, replication, transcription, and recombination [45]. Many studies have suggested that flavonoids might have inhibitory effects on topoisomerase enzymes [46–48]; it was demonstrated that flavonoids such as quercetin, acacetin, apigenin, kaempferol, morin, and luteolin inhibited topoisomerase I-catalyzed DNA relegation [49].
- Suppression of reactive oxygen species (ROS): ROS consist of free radicals (such as superoxide anion and hydroxyl radical) and nonfree radicals such as hydrogen peroxide and hypochlorous acid [50]. ROS can cause oxidative damage to proteins, DNA, and RNA, as well as oxidation of fatty acids in cell membranes which in turn can increase the risk of mutations and promote carcinogenesis [51–54]. However, under physiological conditions, most of the injuries caused by ROS are restored by the body's antioxidant repair system [55]. Flavonoids have been shown to exert a direct scavenging effect on ROS by donating hydrogen atom [56]. They can also suppress the generation of ROS by the inhibition of oxidases such as xanthine oxidase, cyclooxygenase, lipooxygenase, microsomal monooxygenase, NADH oxidase, and glutathione S-transferase, all of which play a crucial role in superoxide anion generation [29, 57]. Furthermore, certain flavonoids can chelate trace metals like iron and copper which results in the reduction of generation of free radicals [58].

*Others.* Flavonoids can also induce apoptosis by a number of mechanisms which include modulation of heat shock proteins expression [59], regulation of signaling pathways [60], release of cytochrome c leading to activation of caspases 9 and 3 [61], decreased expression of Bcl-2 and Bcl-X (L) but overexpression of Bax and Bak, activation of nuclear transcription factor kappaB (NF-*κ*B), stimulation of endonucleases, and downregulation of Mcl-1 protein [62–66].

#### 1.3.2. Cell Cycle Arrest and Inhibition of Proliferation

Cell cycle is a series of molecular events of cell division during which cells increase in size and split to give rise to new daughter cells. For descriptive purposes, it is divided into 4 phases, namely, G_1_ (gap 1 phase), S (synthesis phase), G_2_ (gap 2 phase), and M (mitosis phase). G_1_ and G_2_ are two breaks with variable duration separating the S and M phases; hence, the cycle is usually summarized as G_1-_S_-_G_2_-M [67, 68]. Abnormalities in the execution of normal cell cycle contribute significantly to the development of cancer [69, 70]. Cell cycle is regulated by two key classes of molecules, the cyclin (cyclin A-cyclin T) and the cyclin-dependent protein kinases (CDK 1-CDK 9). CDK-activating kinase (CAK) also referred to as cdk7/cyclin H plays an important role in excitation of CDK/cyclin complexes. The cells usually undergo growth changes as a necessary event before S phase of the cell cycle begins, and c-myc in addition to its role in both cell cycle and cell proliferation is believed to play a key role in this important event marked by an increase in cell size [68, 71, 72]. G_o_ phase which is a resting stage marks the period during which cells stop undergoing cell cycle. The activity of CDKs is also regulated by two families of proteins, the INK4 family made up of p16^INK4a^, p15^INK4b^, p18^INK4c^, and p19^INK4d^ which selectively inhibits CDK4 and CDK6 and the CIP/KIP family which has stimulatory effect on p21^cip1/waf1^ (a potent inhibitor of CDKs), p27^kip1^, and p57^kip2^ [70, 73]. It is now proposed that anticancer agents that target cell cycle could magnificently determine the success of cancer chemotherapy as well as providing clues in finding a complete cure for many tumors; hence, targeting the different stages of cell cycle could play a vital role in the discovery of novel treatment options in cancer treatment [68, 70, 74].

The inhibitory effects of flavonoids such as quercetin and kaempferol on cell cycle in HL-60 and U937 leukaemic cells have been reviewed elsewhere [75]. Resveratrol has been shown to induce overexpression of cyclins A and E with subsequent arrest of HL-60 cells at S/G_2_-phase transition, thus, resulting in accumulation of the cells in the G_1_/S phases [76]. Flavopiridol, which is a novel semisynthetic analog of rohitukine, was shown to inhibit a number of CDKs which include CDK1, CDK2, CDK4, CDK6, and CDK7 [77].

The antiproliferative activity of flavonoids is also believed to play a significant role in preventing tumor progression. A number of flavonoids have been shown to inhibit proliferation in different types of cancer cell lines such as MCF-7 human breast cancer cells [78], HT-29 human colon cancer cells [62], HL-60 leukaemia cell lines [79], and several other cancer cell lines [80]. In addition, several studies have reported that flavonoids such as quercetin, apigenin, curcumin, and nobiletin are capable of suppressing the signal transduction enzymes involved in the control of cell proliferation; such enzymes include protein tyrosine kinase (PTK) [81], protein kinase C (PKC) [82], and phosphoinositide 3-kinase (PIP_3_) [83].

#### 1.3.3. Antioxidant Activity

Oxidative stress as originally defined “refers to an imbalance between oxidants and antioxidants on a cellular or organism level, with potentials to result in injury” [84]. Oxidative stress has been linked with the development of cancer [53, 54]. In addition to the antioxidant effects of flavonoids (direct scavenging effect on ROS, inhibition of oxidases such as xanthine oxidase, cyclooxygenase, lipoxygenase, microsomal monooxygenase, NADH oxidase, and glutathione S-transferase) discussed above, polyphenols are also capable of alleviating oxidative stress induced by nitric oxide (NO) which is believed to cause oxidative damage at high concentrations [58].

#### 1.3.4. Modulation of Multidrug Resistance

Overexpression of P-glycoprotein (Pgp) is associated with multidrug resistance (MDR) in cancer chemotherapy; this resistance can adversely affect the success of treatment of cancer [85]. The major function of Pgp, which is a plasma membrane ATP-binding cassette (ABC) transporter, is extrusion of cytotoxic drugs from the cells at the expense of ATP hydrolysis [85]. As previously reviewed by Ren and colleagues, flavonoids are capable of modulating the Pgp-mediated multidrug resistance via (a) downregulation of expression of MDR-1 gene, (b) direct binding to nucleotide-binding domain (NBD) with high affinity, and (c) suppression of ATPase activity, nucleotide hydrolysis, and energy-dependent drug interaction with transporter-enriched membranes [86]. However, the role of flavonoids still requires further elucidation as contradicting results were obtained when the effects of quercetin, kaempferol, and galangin on different MDR cell lines were studied [58].

#### 1.3.5. Promotion of Cell Differentiation

Cell differentiation is indispensable in normal growth and homeostasis. Leukaemia and some other cancer cells are incapable of differentiating into cells that exhibit mature phenotypic characteristics of nonreplication, thus, remaining in an overwhelming proliferative state with resultant outgrowing of their normal cellular counterparts [87]. Induction of cell differentiation as an alternative for control of abnormal proliferation of cancer cells has received a lot of attention by researchers; it results in elimination of cancer cells thereby reestablishing a normal cellular homeostasis [88, 89]. Commonly encountered flavonoids in propolis such as genistein, apigenin, luteolin, and quercetin have been demonstrated to induce differentiation of HL-60 leukaemia cells into mature granulocytes and monocytes [90]. Constantinou and colleagues have also demonstrated induction of differentiation of K562 leukaemia cell lines into erythroid cells by genistein [91].

#### 1.3.6. Other Signaling Pathways

Modulation of mitogen activated protein kinases (MAPK) has been established as a target for cancer therapy and the role of polyphenols in suppression of these proteins has been extensively reviewed elsewhere [92]. Regulating the activities of MAPKs plays a vital role in the control of gene expression [93].

### 1.4. Brief Description of Various Types of Leukemia Cell Lines Used in the Study of Antileukaemic Effects of Propolis or Its Phenolic Contents

A number of different types of leukemia cell lines representing the various subtypes of leukaemia have been used to examine the antileukaemic property of crude propolis as well as its extracts and its phenolic contents, and they include HL-60, U937, MOLT-4, NALM-6, K562, HL-205, K-562-J, RS4; 11, CEM, NB4, NALM-16, SUP-B15, and REH. We have recently reviewed the origin and characteristics of HL-60, U937, MOLT-4, NALM-6, and K562 cell lines [75]. HL-205 cells are clones of cells isolated from HL-60 cells [94]. K-562-J cells are cell clones isolated from K-562 cells [91]. RS4; 11 was originally isolated from bone marrow of a 32-year-old woman with acute leukemia and it is characterized by t(4; 11) chromosomal aberration [95]. CME, one of the earliest ALL cell lines to be established, was isolated from peripheral blood of a female child [96]. NB4 is a cell line with t(15; 17) chromosomal symbol originally isolated from the marrow of a patient with acute promyelocytic leukemia in relapse [97]. NALM-16 was originally isolated from blood lymphoblasts of a female patient with acute lymphoblastic leukemia in relapse [98]. SUP-B15 was originally grown from bone marrow of an 8-year-old boy with Philadelphia chromosome-positive acute lymphoblastic leukemia in relapse [99]. REH was originally isolated from the peripheral blood of a 15-year-old North African girl with ALL in relapse [100].

Owing to the role of polyphenols as antioxidants, anti-inflammatory, and anticarcinogenic agents as well as in the treatment of heart diseases [86, 101–106], they have received a lot of attention in the recent years [26].

Based on our previous review on the type of polyphenols that have been confirmed to be found in honey, available reports indicate that honey contains slightly different phenolic compounds compared with propolis [75], and at the time of such review, no report was available on the antileukaemic effects of crude honey or its extracts; hence, the present paper reviews the antileukaemic effects of propolis and its extracts as well as those phenolic compounds that have been confirmed to be present in it. It is hopeful that this will shed more light on the potential benefits of combination of honey and propolis in the prevention and/or treatment of malignancies such as leukemia. Table 1 summarizes the active ingredients of the two major types of propolis that possess anticancer and antitumor properties [22, 64, 87, 107–118].

## 2. Effects of Crude Propolis and Its Extracts on Various Leukemia Cell Lines

The effect of a new propolis preparation (CB propolis) isolated from Brazilian propolis on U937 human histiocytic lymphoma cells has been studied by Aso et al. and they found that crude propolis exhibited a dose and time-dependent inhibitory effect on the growth, as well as synthesis of DNA, RNA, and protein in these cells. No obvious antiproliferative effect was observed during the first 8 h of treatment of U937 cells with all the doses of propolis. However, treatment of the U937 cells with 0.015, 0.05, 0.15, and 0.5 *μ*L/mL propolis for 3 days caused growth inhibition in the cells by 9, 25, 44, and 68%, respectively. In a similar fashion, inhibition of synthesis of DNA, RNA, and protein was also observed when the U937 cells were incubated with different doses of propolis. The authors also demonstrated two very important features of apoptosis, namely, chromatin condensation and DNA fragmentation, although, they did not assay individual caspases to determine their role in the apoptotic process; they however suggested that the propolis-induced apoptosis was mediated via caspase pathways [119]. Similar findings have been reported in a different leukemia cell line, HL-60. Another Brazilian propolis extracted with water or ethanol was found to inhibit cell growth which was thought to be as a result of direct cytotoxic effect of propolis and induction of granulocytic differentiation in the HL-60 cells. Further, to establish the role of granulocytic differentiation in induction of apoptosis, features such as nuclear condensation and fragmentation, as well as DNA ladder formation were looked for under fluorescence microscope and they were found to be present, hence, confirming that the apoptosis was partly mediated by granulocytic differentiation of HL-60 cells [120]. These findings were confirmed and even further elaborated by the work of Motomura et al. which provided additional information on the mechanism of propolis-induced apoptosis by examining the expression of antiapoptotic and proapoptotic proteins. They demonstrated that incubation of human leukemic U937 cells with methanolic extract of propolis resulted in decreasing the expression of Bcl-2 (antiapoptotic protein) in a dose-dependent manner; however, no obvious effect was observed on the expression of Bax (a proapoptotic protein). On the basis of this, they suggested that the apoptotic cell death induced by propolis was mediated by mitochondrial mechanism. Furthermore, they observed a dose-dependent decrease in the concentration of procaspase-3 which suggested that there was activation of caspase-3 by propolis. Incubation of the U937 cells with 300 and 500 mg propolis was also demonstrated to cause cell cycle arrest as evident by upregulation of expression of p21 and p27 and downregulation of expression of cyclin A, cyclin B, Cdc2, and Cdk2, all of which are believed to play a key role in cell cycle [121].

Another interesting finding on the mechanism of propolis-induced apoptosis was reported earlier by Gunduz et al. in which they demonstrated the inhibitory effect of ethanolic extract of Manisa (Turkish) propolis on telomerase activity in T-cell lymphoblastic leukemia (CCFR-CEM) cell line by determining the ratio of human telomerase reverse transcriptase (a catalytic subunit of telomerase associated with telomerase activity) which they found to be decreased from 195.56 to 13.29 after treatment of the cell with propolis; they therefore suggested that one of the mechanisms involved in propolis-induced apoptosis may be related to inhibition of telomerase activity [122]. These findings were tested in leukemia cells obtained from bone marrow of paediatric leukemia patients by Cogulu et al. They collected bone marrow from three acute lymphoblastic leukemia (ALL) and one chronic myeloid leukemia (CML) and analyzed the expression of human telomerase reverse transcriptase (hTERT) after treatment with ethanolic extract of Manisa propolis. They found a decrease in the expression of hTERT following incubation of the cells with 60 ng/mL propolis [123]. Korean propolis has also been studied by Eom and colleagues. Most of the above findings were confirmed by this study conducted using HL-60 cells treated with ethanolic extract of the Korean propolis; and they include dose-dependent inhibition of proliferation, induction of apoptosis (evident by DNA fragmentation and formation of DNA ladder), activation of caspase-3 (confirmed by the inhibition of propolis-induced cell death by a caspase-3 inhibitor known as z-DEVD-fmk), and elevated levels of cytosolic cytochrome c. Furthermore, they demonstrated a cleavage of PARP (a protein necessary for DNA repair) in propolis-treated HL-60 cells; hence, they concluded that caspase-3 activation played a significant role in propolis-induced apoptosis in HL-60 cells and that the cell death was mediated by mitochondrial pathway [124].

Brazilian propolis has been well investigated in the recent years and based on its physicochemical features is classified into 13 groups [125]. The effect of ethanolic extracts of propolis group 12 (G12) also known as green propolis and propolis group 13 (G13) also termed red propolis was assessed in K562, HL-60, NB4, Ramos human Burkitt lymphoma, Raji human Burkitt lymphoma, Nalm 16, Nalm 6, RS4, B15, and REH cell lines and it was observed that both extracts from red and green propolis were cytotoxic to all the tested cells with the red propolis being more effective compared with the green propolis [125].

## 3. Commonly Studied Phenolic Contents of Propolis

Propolis contains a variety of polyphenols; however not all of them have been studied extensively.  Table 2 shows the most commonly studied classes of phenolic contents of propolis with particular emphasis to those that have been shown to possess antileukaemic effects [24, 26, 120, 126–131]. The antileukaemic activity of quercetin, kaempferol, galangin, myricetin, apigenin, acacetin, chrysin, and luteolin has been previously discussed by our team who reported that these compounds exert antileukaemic activity through various mechanisms which include cell cycle arrest, inhibition of growth and proliferation, and induction of apoptosis and differentiation in various leukaemia cell lines [75]. However, the antileukaemic activity of genistein and cinnamic acid derivatives like baccharin, drupanin, and artepillin C which has not been previously reviewed is discussed below.  Table 2 summarizes the mechanisms of action of propolis and its phenolic constituents on various types of leukaemia cell lines.

## 4. Antileukaemic Role of Phenolic Contents of Propolis 

### 4.1. Baccharin and Drupanin

Baccharin ((*E*)-3-prenyl-4-(2, 3-dihydrocinnamoyloxy) cinnamic acid) and drupanin ((*E*)-3-prenyl-4-hydroxycinnamic acid) are cinnamic acid derivatives reported to be found in ethanol extract of Brazilian propolis. Akao and colleagues have examined the anticancer properties of these compounds in human leukemia (HL-60) cells. They observed growth inhibition in these cells after incubation with a dose of 150 *μ*M of these compounds. Nuclear condensation and fragmentation with typical DNA ladder formation were also observed following 24 h treatment of these cells with the compounds; hence, it was concluded that baccharin and drupanin arrested HL-60 cells growth through induction of apoptosis [132].

### 4.2. Genistein

A number of studies have examined the antileukaemic effects of genistein and this isoflavone has been found to be present in a number of propolis extracts of different origins that include Argentina, Italy, Spain, and Ethiopia [133]. The anticancer property of genistein has been studied by Constantinou and coworkers using leukaemia cell lines. They reported that treatment of human myeloid HL-205 and erythroid K-562-J leukemia cells for 6 days with different doses of genistein resulted in a time- and dose-dependent inhibition of cell growth and cellular differentiation; the latter was evident by the increased percentage of the cells reacting with OKM 1 monoclonal antibody, positive staining for NSE (a usual feature of monocytes), and expression of NBT dye reduction (a symbol of granulocytic maturation) in the leukaemia cells. Although over 95% of the HL-60 cells exhibited the reaction with OKM I antibody, the percentage of cells observed to show lineage-specific markers (monocytic or granulocytic) did not surpass 50%; hence the authors suggested that genistein might have a dual biochemical effect in this circumstance, so that on the one hand it inhibited Topo II which could lead to differentiation into one phenotype and on the other hand it inhibited tyrosine kinase which could result in differentiation of the other phenotype [91]. In a similar report, Traganos and colleagues examined the antileukaemic activity of genistein on HL-60 and MOLT-4 cells. They demonstrated that genistein induced a dose- and time-dependent cell cycle arrest at G_2_/M and S phases in both HL-60 and MOLT-4 cells [134]. Similar studies later confirmed the antiproliferative activity of genistein in Jurkat cells [135] and HL-60 cells [79].

Li and coworkers have recently tested the effects of genistein on ALL, lymphoma, and multiple myeloma cell lines, and they demonstrated a dose- and time-dependent inhibition of all the tested cells with RS4; 11 being the most sensitive. They further revealed that treatment of RS4; 11 cell lines with genistein repressed antiapoptotic signaling proteins by demonstrating increased cleavage products of caspases 3, 7, and 9 and PARP as well as downregulation of expression of cIAP and Bcl-2 proteins in a dose-dependent pattern. It was also found that genistein induced late apoptotic processes by demonstrating an increased population of cells with positive staining for brominated deoxyuridine triphosphate nucleotide (a nucleotide that attaches to the 3′-hydroxyl ends of DNA fragments in apoptotic cells). Genistein was also demonstrated to cause cell cycle arrest at G_2_/M phase as well as accumulation of cyclin B1 and upregulation of P21 and P53 genes in the tested ALL cell lines [136].

### 4.3. Artepillin C

Artepillin C (3,5-diprenyl-4-hydroxycinnamic acid) has been reported to be found in ethanolic extract of Brazilian green propolis [137]. Kimoto and coworkers have assessed its antitumour activity on different phenotypes of human leukaemia cell lines such as lymphocytic leukaemia (both cell lines of T-cell and B-cell), myeloid and monocytic leukaemia, and nonlymphoid nonmyeloid leukaemia cell lines and found that it induced apoptotic cell death (marked by appearance of apoptotic bodies and DNA fragmentation) in all the tested cells; and they suggested that this effect may be partly associated with upregulation of Fas expression and loss of mitochondrial membrane potential [138].

## 5. Conclusions 

Propolis and its phenolic contents have been shown to have antileukaemic activities which are achieved through a number of mechanisms that include induction of apoptosis and differentiation, cell cycle arrest, and inhibition of growth and proliferation in various types of leukemia cell lines. This suggests that propolis and its polyphenols components may be used as effective adjuncts to chemotherapy in the treatment of cancers. However, the fact that the phenolic contents of propolis vary depending on their botanical and geographical origin signifies the need for further research to quantify individual polyphenols from different botanical sources as well as from different geographical regions. There is also the need to carry out* in vivo* and epidemiological studies as well as clinical trials in order to fully evaluate the potential role of this important bee product in the discovery of novel agents for both treatment and prevention of leukemia and other haematological malignancies. Furthermore, due to paucity of documented data on bioavailability and toxicity of propolis, researches on local propolis are necessary. It is also suggested that the use of propolis in combination with honey for prevention and treatment of many ailments including malignancies as practiced traditionally should be further elucidated.

## Figures and Tables

**Table 1:** Biologically active ingredients in poplar and *Baccharis *propolis.

Biological activity	Propolis type, active ingredients
Anticancer and antitumour	***Baccharis***: artepillin C, baccharin, drupanin, cinnamic acid derivatives, prenylated p-coumaric acids, clerodane diterpenes, benzofuranes, and caffeoylquinic acid derivatives
	**Poplar**: caffeic acid, caffeic acid phenylethyl ester, apigenin, quercetin, genistein, galangin, luteolin, fisetin, ferulic acid, chrysin, acacetin, p-hydroxy-benzoic acid, p-methoxy-benzoic acid, gallic acid, and vanillic acid

**Table 2:** Summary of mechanisms of action of propolis and its commonly studied phenolic contents.

Propolis/phenolic compound	Cell type	Mechanisms of action	References
Propolis	U937 HL-60 K562	Induction of apoptosis, inhibition of growth and proliferation, and cell cycle arrest Direct cytotoxicity and induction of differentiation and apoptosis Induction of apoptosis	[119, 121] [120] [125]

Quercetin*	HL-60 K562 U937	Cell cycle arrest and inhibition of growth and proliferation Induction of apoptosis and differentiation Induction of apoptosis and cell cycle arrest	[75] [75] [75]

Kaempferol*	HL-60	Cell cycle arrest and growth inhibition	[75]
Galangin*	HL-60	Inhibition of proliferation and induction of apoptosis	[75]
Myricetin*	HL-60	Induction of apoptosis	[75]

Apigenin*	HL-60 THP U937	Induction of apoptosis Induction of apoptosis Induction of apoptosis	[75] [75] [75]

Acacetin*	Jurkat cells	Growth inhibition and induction of apoptosis	[75]
Chrysin*	U937	Induction of apoptosis	[75]
Luteolin*	HL-60	Growth inhibition and induction of apoptosis	[75]
Baccharin and drupanin	HL-60	Growth inhibition and induction of apoptosis	[132]

Genistein	K562 HL-60 MOLT-4 RS4; 11	Inhibition of growth and induction of apoptosis Cell cycle arrest Cell cycle arrest Induction of apoptosis and cell cycle arrest	[91] [91, 134] [134]

Artepillin C	T-cell, B-cell	Induction of apoptosis	[138]

*Previously reviewed by our team [75].
